# Interventional Radiology’s Osteoid Osteoma Management: Percutaneous Thermal Ablation

**DOI:** 10.3390/jcm11030723

**Published:** 2022-01-29

**Authors:** Giampaolo Bianchi, Luigi Zugaro, Pierpaolo Palumbo, Roberto Candelari, Enrico Paci, Chiara Floridi, Andrea Giovagnoni

**Affiliations:** 1 Diagnostic and Interventional Radiology, SS. Filippo e Nicola Hospital, 67051 Avezzano, Italy; 2 Department of Biotechnological and Applied Clinical Sciences, University of L’Aquila, 67100 L’Aquila, Italy; luigi.zugaro@virgilio.it (L.Z.); palumbopierpaolo89@gmail.com (P.P.); 3 Interventional Radiology, Department of Radiology, Az. Osp-Univ OORR-Torrette, 60100 Ancona, Italy; r.candelari@ospedaliriuniti.marche.it (R.C.); enrico.paci@ospedaliriuniti.marche.it (E.P.); 4 Department of Radiology, University Hospital “Umberto I-Lancisi-Salesi”, 60100 Ancona, Italy; chiara.floridi@ospedaliriuniti.marche.it; 5 Department of Radiological Sciences, Ospedali Riuniti, Marche Polytechnic University, 60100 Ancona, Italy; a.giovagnoni@univpm.it

**Keywords:** osteoid, osteoma, oblation techniques, percutaneous, magnetic resonance imaging, interventional, radiology

## Abstract

Osteoid osteoma (OO) is one of the most common benign bone tumors with specific clinical and radiological characteristics. Analgesic therapy and surgical treatment have been considered the only therapy for a long time. Recently, safe and effective new therapeutic options have been introduced, among which percutaneous thermal ablation techniques. This review aims to describe the recent updates in the field of percutaneous thermal ablation techniques in the treatment of OO, assessing the outcomes in terms of efficacy, complications, and recurrence rate.

## 1. Introduction

Osteoid osteomas (OO) are common benign bone tumors, representing 3% of all primary bone tumors [1]. OO typically affects the diaphysis or metaphysis of long bones in the lower limb. OO can be classified in cortical, medullary, and subperiosteal OO can be classified in cortical, medullary, and subperiosteal.

Cortical OO is the most common type; it generally presents with a typical central nidus surrounded by peripheral sclerosis. Medullary and subperiosteal OO are less frequent and often have less peripheral bone sclerosis [2].

Clinical presentation often includes bone pain which is stronger at night and is associated with a significant pain relief after the use of nonsteroidal anti-inflammatory drugs (NSAIDs). Pain impairs the quality of life of the patients as it is usually associated to sleep deprivation. [3]. The gold standard treatment has been represented for a long time by surgical resection for those patients suffering from severe pain, who did not benefit from conservative treatment or could not tolerate long-term NSAIDs therapy [2].

The treatment of OO has changed in the last decades, and the percutaneous thermal ablation techniques are now considered as the gold standard due to their feasibility, safety, and effectiveness [4]. Furthermore, another advantage is represented by shorter hospitalization time compared to surgical excision. All thermal ablation procedures generate image-guided coagulation necrosis in the target tissue using special tools (electrodes, antennas, probes) placed in the tumour. The extension of the area of coagulation necrosis is related to important factors, such as the quantity of applied energy, local tissue interactions, and technical problems (including losses of heating or cooling effects) [5].

The percutaneous thermal ablation techniques reported in literature include radiofrequency thermal ablation (RFA), interstitial laser ablation (ILA), cryoablation (CA), and microwave ablation (MW). RFA is the most attractive technique because it is effective, efficient, and safe. ILA is a relatively user-friendly low-cost alternative. CA has the advantage to show in real-time the ice-ball forming in the target area. This maximizes safety of the technique. CA is used to relieve pain and may bring immunotherapeutic benefits. MWA has become attractive in the last years due to its potential advantages over RFA represented by the possibility to reach higher temperatures within the target lesion, being less sensitive to changes in tissue composition.

Aim of this review of the literature was to describe the management of osteoid osteoma (OO) with interventional radiology techniques and to assess the results in terms of efficacy, complications and recurrence rates for each technique [6].

## 2. Radiofrequency Ablation (RFA)

During the last 30 years, RFA has progressively replaced the surgical treatment and, due to its safety and effectiveness [7,8,9,10,11,12], became the gold standard for the treatment of osteoid osteoma (OO).

During the procedure, an ablation electrode is inserted inside the OO nidus under CT guidance. The lesion is then heated for 5–6 min, usually at 90 °C.

Most patients undergo conscious sedation and spinal anesthesia; less frequently, general anesthesia is needed. Pain control is usually not obtained with local anesthesia alone [3,7].

Currently, different ablation techniques are available; each with different temperature settings and treatment duration.

For example, Martel J. et al. treated 38 patients affected by OO by using RFA under regional or general anesthesia and illustrated an RF ablation method using an exposed cool-tip electrode (1 cm), rather than the traditional non-cooled 5 mm electrode. Follow-up duration was set up at 3 days; 3 weeks; and 3, 6, 12, and 24 months. The authors reported clinical success in 97% of the treated patients, defined as pain reduction assessed with the visual analogue scale (VAS score) before and after the procedure. No symptoms were reported in all patients during the follow-up time. In one case, nidus destruction was not complete and a further RFA procedure was necessary due to incorrect placement of the electrode. Complication rate was low with only two minor complications reported [13]. Another study described a different RFA technique, performed using a cool-tip electrode without the cooling system in a group of 17 patients; coagulative necrosis was obtained heating the lesion for 4 or 5 min at the temperature of 90°C. The procedure was considered technically successful when the electrodes were correctly placed into the nidus and ablation performed for at least 4 min after the target temperature was reached. Another outcome, such as clinical success, was assessed by evaluating post-procedural pain relief. The results showed an overall clinical success of 88.2%, with all procedures considered technically successful; only 2 patients experienced pain recurrence and one patient underwent a second ablation procedure with RF. In this study, the number of complications was also very low, with only two patients experiencing minor complications (pes equinus contracture and skin burn) [14]. In a different ablation protocol proposed by other authors, the temperature was initially set up at 90 °C with a subsequent plateau (2 min) at 60 °C. RF ablation was then performed for more than 15 min [15,16,17,18].

In all these studies, the success rate was very high (>90%). One author reported appreciable success rates using this RFA protocol (from 79 to 98%), with a significant reduction of recurrences (from 21% to 2%). Moreover, the complication rate dropped from 5.9% to 0.2% (*p* < 0.001) when compared to the direct heating of the nidus at 90 °C, and ablation lasting 4 min. Larger lesions were treated with multiple ablations performed during the same session, since all patients with large and multiform lesions had recurrence when treated with one ablation alone [17].

Akhlaghpoor et al. combined RFA and alcohol ablation during the same session.

Firstly, using a coaxial drill system, the cortical bone was drilled till the tip reached the center of the nidus. The drill was removed, and radiofrequency ablation was performed using a single cool tip; subsequently, a straight rigid electrode with an active tip (1cm) was placed in the center of the nidus. The tip of the electrode reached a temperature of 90 °C, maintained at least for 6 min and monitored by the manual mode control of the generator during the procedure. At the end of the ablation cycle, the RF needle was removed, and an injection of absolute ethanol was then performed into the nidus using a 15 cm, 20 G Chiba [19].

The study included 54 patients, and immediate pain relief was reported in 52 of them; recurrences were observed in 2 patients, who were submitted to a second combined ablative procedure leading to complete pain relief [19].

In 2020, a meta-analysis published by Lindquester et al. analyzed all the interventional radiology’s techniques available for the therapy of OO and performed a pooled analysis with subset evaluation of intra-articular and spinal tumors [4]. The authors retrospectively analyzed 36 studies, 32 of which (88.9%) evaluated RFA; 3 (8.3%) analyzed cryoablation (CA), and 1 (2.8%) microwave (MW) ablation. For each study, they assessed clinical success, failures, recurrences, and success rates [4,7].

Success was defined as pain-free time without recurrence during the entire follow-up period. Clinical failures were defined as persistence of pain after treatment, and recurrences as symptoms relapse after a pain-free period. The success rate was defined as the percentage of cases without technical failures.

In this study, the overall success rate was 91.9% (95% confidence interval 91–93%), with no significant difference (*p* = 0.92) in terms of success rate between RFA and cryoablation (91.9% for RFA and 91.6% for cryoablation, respectively). The success rate for MW was 100%. Technical failures occurred in 0.3% (95% confidence interval, ranging between 0.2–0.7%) and complications were seen in 2.5% of the procedures (95% CI, ranging from 1.9 to 3.3%).

A very important result of this meta-analysis regards the statistically similar outcomes of RFA and cryoablation in the treatment of osteoid osteoma [4]. CA might have additional benefits in comparison with RF, as described in a study that assessed clinical outcomes of different techniques used for treatment of bone metastases, showing a significant reduction in pain, better medical therapy management with a decrease in opioid drugs use and shorter hospitalization times in patients treated by CA in comparison with RFA. This result might also apply to OO treatment [20], although further studies are needed to prove this evidence.

In a review published by Masciocchi et al., the authors compared outcomes in pain relief and motor functional recovery with osteoid osteoma treated by magnetic resonance-guided focused ultrasound surgery (MRgFUS) or radiofrequency ablation (RFA), using a propensity score matching study design. In this study, 32 patients affected by OO were included. Fifteen patients underwent RFA and 15 were treated with MRgFUS. 

MRgFUS uses a focused ultrasound beam to induce coagulation necrosis of the target tissue. The results showed that 94% of patients treated with MRgFUS and 100% of those submitted to RFA experienced CR 12 weeks after treatment, with no significant difference between both groups. The improvement in pain control following MRgFUS or RFA was associated to improved motor functional recovery. The treatment failure rate was 6.6% in the MRgFUS group and 0% in the RFA group with no major complications observed in the two groups of people treated by different ablation techniques [21].

A group of authors reviewed the literature to assess the efficacy of percutaneous thermal ablation procedures in the treatment of OO, with a particular focus on recurrences, to understand whether the latter are due to true osteoid osteoma relapse or incomplete treatments. Also, safety and efficacy during long-term follow-up in an extremely large cohort of patients were assessed [22]. 

A systematic review analyzed 27 clinical trials of OO treated by the different percutaneous thermal techniques to detect the causes of recurrences, and and enlist the occurred complications and the reported methods to prevent them.

Radiofrequency ablation was evaluated in 23 articles and interstitial laser ablation (ILA) was carried out in 3 trials, while only 1 focused on combined RF-ILA treatment. The reported success rate was very high (90–100%) and the complication rate was 2%, with 5% of patients who experienced uncomplete response to the treatment or recurrence. According to the authors, RFA represents the best therapeutic option for treatment of patients affected by OO compared to surgical therapy. Among the twenty-seven clinical trials evaluated, 23 described RFA, 3 interstitial laser ablation (ILA), and 1 combined RF-ILA. The reported success rate was 90–100%, and the complication rate was low (2%) with 5% of patients, who did not successfully respond to the treatment or experienced recurrence. This study confirms that percutaneous thermal ablation remains the treatment of choice compared to surgery, with the prevalence of RFA over the other techniques. According to the authors, however, treatment failure and recurrence causes were not accurately assessed, suggesting that a deeper analysis of these factors could have led to statistically significant guidelines for clinical practice. The lower risk of recurrence was related to a longer ablation period [22].

A systematic review published by Tordjman M. et al. in 2020 evaluated 69 articles and put a particular focus on treatment failures connected with RFA treatment, assessing the factors associated with it. Treatment failure was defined in terms of recurrences, persistency of symptoms, and complications. The authors investigated on whether such factors as lesion location, ablation time, and patient age may affect the recurrence rate. The results of this study confirmed that longer ablation times (>7 min) were associated to lower treatment failure rates, while other elements like age and OO location did not show a statistically significant association with recurrence rate [23].

Another study tried to identify the variables associated to symptomatic recurrence of OOs by studying 71 patients, who underwent CT-guided RFA [24]. Ten patients had symptomatic recurrence after treatment in a median follow-up duration of 29 months (ranging between 10 and 90 months). All patients who experienced a relapse of symptoms underwent a successful second ablation treatment. According to the results of this trial, such variables as age <13 years, female gender, and “eccentricity index” (EI) ≥3 may be considered risk factors for symptomatic recurrence of OO after percutaneous treatment with RF [24]. EI is considered as the ratio obtained by dividing the greatest maximum length by the lowest maximum length from all anatomic planes [25].

With reference to the follow-up after percutaneous thermal ablation treatment of OO, usually, ablation is considered clinically successful when there is pain regression; in the radiological follow-up after RFA treatment, MRI plays a central role as described in literature. 

Radiologically, the procedure is considered effective and successful when, in the period following the thermal ablation, the nidus of OO is replaced by a necrotic core [26]. In this phase, associated signs of inflammation of the adjacent bone may also be observed, as well as hyperemia and edema with ring-like appearance around the treated lesion [26]. In a recent review, Arrigoni et al. retrospectively evaluated the imaging outcomes of the follow-up of successful minimally invasive treatments of OO.

**Figure 1:**
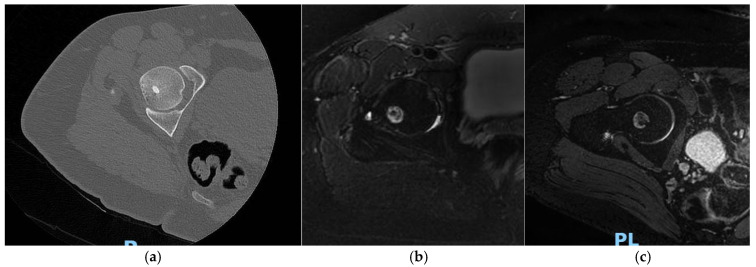
Osteoid osteoma of the femoral head: CT (**a**) and MRI T2W image with fat saturation (**b**): perilesional reaction without marrow bone edema; (**c**) after 6 months, the lesion is dimensionally stable.

The aim of their study was to define the main findings on the imaging that can best describe the regular evolution of these types of treatments. Four findings were considered by the authors as particularly relevant (Figure 1): (1) bone marrow edema; (2) reactive phenomena (perilesional inflammatory reaction for extra-articular lesions or synovial reaction for intra-articular ones); (3) bone remodelling (disappearance of the nidus and bone healing); (4) ring sign (granulation tissue around the treated nidus). These findings were assessed using MRI and CT with a follow-up study that lasted up to 24 months. Thirty-four patients were evaluated; during the follow-up period, the authors noticed a consistent reduction of all inflammatory signs such as, for example, the bone marrow edema, which was significantly reduced. Furthermore, synovitis disappeared in 53% of patients and was reduced in the other 46%; the perilesional inflammatory reaction was significantly reduced in 76% of the patients and disappeared in 24% [27].

After one month from the treatment, the site of the nidus is usually replaced by cicatricial/fibrotic tissue [28]. Progressively, MRI follow-up exams show a reduction of the perilesional bone marrow edema. Contrast-enhanced MRI is important in the diagnosis of OO, since enhancement of the nidus is a pathognomonic sign. MRI is also a crucial tool in the early identification of possible post-treatment disease relapses [29,30]. Some studies support this last suggestion, for example Mahnken et al. studied 20 patients and demonstrated that contrast enhancement on T1-weighted MRI imaging seems to be predictive of treatment failure after radiofrequency ablation for treatment of OO [30]. MRI is also useful to assess post-procedural complications, for example adjacent soft tissues lesions or osteomyelitis [31]. The results of percutaneous RFA for treatment of OO, as reported in the literature studies, are summarized in the Table 1.

## 3. Microwave Ablation (MWA)

Microwave ablation (MWA) causes coagulation necrosis of the target lesion with high intratumoral temperature produced by the perturbation of water molecules exposed to the electromagnetic field. For this reason, microwaves produce higher intralesional temperatures than, for example, RFA, resulting in larger and more homogeneous ablation volumes, as they are not sensitive to impedance. 

The above-mentioned advantages can be obtained by inserting one needle, that, theoretically, may not even be placed precisely in the centre of the nidus [32].

This is the reason why MWA is particularly appreciated in the treatment of hidden lesions or located in a difficult anatomical site.

Many studies evaluate the use of MWA on lesions located in the liver, lungs, kidneys, or adrenal glands. However, there are scarce trials assessing the safety of MWA treatment of benign bone lesions, because the latter are often smaller than 1 cm and require short ablation volumes that increase the risk to damage non-target surrounding tissues [33].

In a recent review, Cazzato et al. state that MWA is effective in providing short- (1 month) and mid-term (4–6 months) pain relief after treating painful OO and bone metastases. In their opinion, however, further studies are needed to collect data about its safety because thermal energy might induce some complications including secondary fractures [34].

In 2013, Basile et al. published an article to evaluate the feasibility of MWA in the treatment of epiphyseal OOs. The authors evaluated 7 patients, who underwent CT-guided thermal ablation using microwaves for treatment of OO located in the long bone epiphysis, far away from nerves and vascular structures. Technical and clinical success were evaluated. A procedure was considered technically successful when the antenna was well placed into the nidus; clinical success ensured a post-procedural complete remission of pain evaluated by using the visual analogue scale (VAS) of <1 without medication. Clinical follow-up was performed evaluating VAS score at 1 day, 1 week, and 1, 3, and 6 months after MWA. Radiological follow-up was performed using MRI immediately after the treatment, after 1 month, and in any case of recurrence. The follow-up duration was 5–13 months. In this study, 100% of patients had neither symptomatology after the treatment nor major complications. Although the sample of patients was small, this study showed how MWA can be performed with excellent results without complications in OOs located away from vascular structures and nerves (epiphysis of long bones) [33].

Similarly, another study assessed the efficacy of MWA evaluating a group of 10 patients treated by MWA under CT guidance. All patients had no symptoms after 1 week from the treatment, with 100% technical success and no complications reported [35].

The largest cohort of patients presenting OO and treated with MWA was described by Rinzler et al. The authors assessed the feasibility and clinical efficacy of MWA in OO treatment in the paediatric population [36].

In this trial, the authors assessed the clinical efficacy of MWA in OO treatment in the pediatric population by evaluating 24 patients treated by MWA. Three ablation cycles were made reaching the goal temperature of 90 °C, with 30 s cooling period between cycles. Clinical follow-up after treatment was performed at 1 week and 1 month. Technical success was defined when the MW antenna was placed at the distal margin of the nidus of the lesion and the target temperature of 90 °C was reached. Clinical success was defined as the complete absence of symptoms after the procedure without pain medication, at 1-month follow-up. In this study, clinical success was 100%, and the complication rate was 17%, although the limit was the short duration of the follow-up period (1 month) [36].

The feasibility of MWA was also assessed by other authors who prospectively evaluated 13 patients treated by CT-guided MWA. Clinical success was defined as the complete absence of symptoms in patients after the treatment, assessed using a numeric pain rating scale (NRS). Also, the authors performed a contrast-enhanced MRI to evaluate nidus vascularization and the necrosis area induced by the ablation. The reported success rate was 92.3% with only 1 case of treatment failure [32].

An interesting comparison between RFA and MWA for treatment of OOs was published in 2020 by Reis et al. [37]. The safety and efficacy of RFA and MWA were assessed and compared. In this retrospective study, the patients were divided into 2 groups based on the treatment they had received (15 patients = RFA; 15 patients = MWA). Technical success was 100% for MW and 83% for RF, respectively; each group presented 1 case of recurrence that needed repeat treatment (after 25 months in a patient treated by MWA and after 22 months post-RFA). In the group of patients treated by MWA, only one case of major complication was reported: second-degree skin burns at the access site evolved in cellulitis that needed treatment with intravenous administration of antibiotics in the hospital. In one case only, a minor and self-resolved complication was reported (numbness in the treated area). In the group of patients treated by RFA, no major complications were observed, with just one minor complication reported in a patient who suffered low-back pain due to sciatic nerve irritation after ablation of OO located in the ischium [37]. In general, no statistical differences were noted between the group treated by RFA and the one treated by MWA, with a comparable long-term recurrence rate (*p* = 1.0) and complication rates (*p* = 0.60) following RFA and MWA [36]. In conclusion, more studies on a larger cohort of patients and longer follow-up periods (>6 months) are needed to confirm the high clinical and technical success rates and few complication rates described in literature (Table 2). 

## 4. Cryoablation (CA)

The use of cryoablation (CA) in the treatment of OO was first described in 2000 by Skjeldal et al., who treated a lesion located in the ischial bone. Follow-up after the treatment showed good clinical success after 1 year [38]. In the last years, CA has become attractive as an alternative option for the treatment of OOs due to the constant increase in literature of data concerning its safety and effectiveness. During CA, the ablated volume appears as a hypodense area (ice ball). This makes the procedure safe and precise because the real-time monitoring of the ice ball helps avoid iatrogenic damage to the non-target surrounding structures. This is particularly important in the treatment of lesions placed near nerves and vessels. 

Wu et al. wrote an article with the purpose to assess the safety and efficacy of CT-guided CA in a group of six pediatric patients affected by OO. Cryoablation protocol included two freezing-thawing cycles and was performed using probes with a diameter of 2.0 mm (14 G). During a follow-up time of 28 months, clinical and technical success were evaluated. Technical success was obtained in all patients; no major complications were observed. Minor complications, such as fever, occurred the day after the procedure in one case only. Pain intensity, evaluated using a VAS score pre- and post-treatment, significantly decreased in all patients. No cases of recurrence were observed [37]. A similar study investigated the safety and efficacy of CT-guided percutaneous CA for the treatment of OOs in adults, retrospectively evaluating 10 adult patients affected by painful OO, treated by CT-guided percutaneous CA. Reported clinical and technical success rates were 100%; pain intensity assessed by a VAS score significantly decreased after treatment; and no recurrences or any major or minor complications were observed [39]. Another trial assessed the feasibility of CA in a group of 21 patients treated by percutaneous cryoablation for treatment of OO. Pain intensity was evaluated using a visual analogue scale (VAS) before and after the procedure. The results reported an overall clinical success rate of 95.2% with no major complications observed. Three cases of minor complications were observed (mild skin burns and soft tissue swelling) [40]. Clinical efficacy of CA in the treatment of OO in a paediatric and adolescent group of patients was published by Whitmore et al. Twenty-nine patients affected by OO were treated with 100% technical success and with high safety, as only six cases of minor complications were reported, due to an inappropriate probe position; however, no major complications were observed [41]. Cryoablation is a safe and effective percutaneous treatment option, with a low complication rate and the advantage to have a real-time control of the ablation area showing on CT the characteristic hypodense “ice ball sign”. (Table 3)

## 5. Interstitial Laser Ablation (ILA)

Interstitial laser ablation (ILA) is a valid alternative to percutaneous radiofrequency ablation (RFA). Technically, it requires a laser generator used at low energy levels and optical fiber to deliver light energy to the target lesion with subsequent conversion into heat energy. High temperature provokes cellular necrosis in the lesion. This makes it possible to estimate the necrotic area according to the water content of tissues, with a necrosis threshold represented by exposure to 50 °C for 30 s. ILA has some advantages in comparison with RFA, as reported by some authors. Here, there is no interference with pacemakers or electronic devices as no current flows are elicited in patient’s body. Also, ILA is cheaper than RFA [42]. A review of the literature showed an overall success rate between 94% and 100% for the treatment of OO using ILA [43,44]. Tsoumakidou et al. retrospectively evaluated the safety and efficacy of CT or fluoroscopic-guided laser photocoagulation for the treatment of spinal OO, in a group of 58 patients with spinal OO). Target lesions located near nerves were treated by ILA to avoid the risks brought about by other percutaneous thermal ablation techniques. The technical success rate was 100%, the clinical relapses were very low (5.3%), and no major complications were reported. All recurrences were treated again percutaneously. Gangi et al. were the first authors to describe cases of OOs treated with photocoagulation. They evaluated the effectiveness and safety of ILA in a large cohort of patients (114 pts) treated over 10 years (1994–2004), reporting an overall success rate of 99%. Six cases of recurrences were observed during the follow-up period (58.5 months). All recurrences were treated again with ILA [45].

Roqueplan et al. assessed the efficacy and safety of ILA, compared to percutaneous resection in a group of 100 patients. The authors reported a success rate of approximately 94% in a 24-month follow-up. The success rate was 95% in the group of patients treated by percutaneous resection (*n* = 26). The group of patients treated by ILA reported one case of technical failure and three cases of recurrences with one patient who did not have pain alleviation after treatment. Treatments were repeated in all recurrences, but failure was observed again in 2 of them [43]. The authors observed that those clinical failures were more frequent in patients < 16 years old. One major complication was reported in a patient who had an iatrogenic lesion of the peroneal nerve; three minor complications occurred, including a case of post-treatment infection, hematoma, and tendinitis. In the other group that underwent percutaneous resection, the complication rate was 12% with three minor complications reported.

An article published by Etienne et al. assessed the efficacy of ILA for the treatment of OOs and aimed to identify the factors leading to technical failure. The authors retrospectively evaluated 35 patients, with clinical and radiological evidence of OO, who underwent ILA. Technical and clinical success, failures, recurrence, and complications were evaluated. Technical success was defined as the correct position of laser within the nidus; clinical success was defined as disappearance of pain 1 month after the procedure; failure as persistence of pain 1 month after treatment; recurrence as persistence of post-procedural symptoms after more than 1 month after treatment following initial pain relief.

The technical success rate was 100% with a mean follow-up duration of 40 months, while the clinical success rate was 94%. The recurrence rate was 6%. All patients, still painful after the treatment, underwent a second procedure.

The complication rate in this study was approximately 5.4% with no major complications. Four minor complications were reported. In one case, the needle got broken during the procedure. Other patients experienced bone lacuna, patellar enthesopathy, and skin burns in the treatment site [42]. A prospective study written by Witt et al. aimed to assess pain intensity before and after treatment in 23 patients treated by ILA for osteoid osteoma. The median follow-up time was 15 months, during which 3 cases of minor complications were observed [46]. Similarly, a study involving a larger cohort of pediatric patients (*n* = 68, mean age 12.1 years) had a 98% success rate with a mean follow-up duration around 83 months. Recurrence occurred in 5 patients in the immediate period following the treatment, due to non-optimal ablation of OO nidus. No complications were related to the procedure, which was demonstrated to be effective and safe even in children [46]. Some authors focused on the safety and effectiveness of ILA in the treatment of vertebral OOs. ILA reported the same low recurrence and complication rate as non-spinal OO treated by percutaneous thermal ablation [44,47].

In addition, Gangi et al. described a successful treatment using photocoagulation in 12 cases of spinal OO without any reported complications [45]. In conclusion, the literature suggests that ILA is a valid and safe alternative to percutaneous thermal ablations using radiofrequency although further data are necessary to validate results of ILA treatment of OOs located in the spine. (Table 4)

## 6. Conclusions

Percutaneous thermal ablation techniques are a safe and effective therapeutic option in the treatment of osteoid osteomas (OO). Among the currently available ablation techniques, RFA is considered the gold standard due to the possibility to perform a preliminary biopsy, whose utility is however still controversial [48]. Radiofrequency ablation (RFA) is the most used technique, probably due to the minor costs in comparison with the other minimally invasive techniques, although not many studies have focused so far on the differences in terms of costs between all the percutaneous ablation methods in the literature. RFA and cryoablation (CA) have statistically similar outcomes in terms of technical success, clinical efficacy, and low recurrence and complications rate. CA is the only technique allowing real-time visualization of the ablated volume (ice ball sign), which maximizes treatment safety. Furthermore, CA shows promising results in patients submitted to treatment of bone metastases in terms of better medical therapy management, pain relief, and subsequent improvement of life quality. [20]. CA brings lower risks of permanent nerve damage when the lesions are located near neural structures. There is even evidence of nerve regeneration after damage caused by cryoablation [49].

MWA allows larger and more homogeneous ablation volumes than RFA, since microwaves are not sensitive to impedance. Another advantage is that the technique is less time-consuming [36]. Microwaves ablation could be used in hidden lesions because there is no need to place the needle exactly at the centre of the nidus [32]. Despite the promising results, however, a few studies are available in the literature, statistically comparing the efficacy of MWA with the other ablation techniques [4]. There are different opinions about the employed MWA ablation protocols described in literature. Some authors [32,50] use amounts of energy that seem excessive for OO ablation. From experience, the effective and complete destruction of a nidus of OO measuring <10 mm is obtained with no more than 1.2 KJ [34]. As regards interstitial laser ablation (ILA), it has also demonstrated good outcomes in the treatment of OO and represents a safe and valid ablation technique, with an overall success rate ranging from 94 to 100%, as reported in literature [42,43,44,45,46,51,52].

## Figures and Tables

**Table 1:** RFA treatment.

Author	N. of Procedures	Success Rate	N. of Recurrences	N. of Complications	Follow-Up (Months)
Martel	38	97%	1	2 (minor)	24
Miyazaki	17	88.2%	2	2 (minor)	1–6 months
Albisjnni	61	93.55%	4	1 (minor)	41.5
Albisinni	27	96.43%	1	2 (minor)	67.4
Rimondi	557	99.6%	24	2 (Major), 3 (minor)	12
Rimondi	97	97.3%	15	1 (Major), 1 (minor)	12
Akhlaghpoor	54	100%	2	2 (minor)	30.5
Baal	71	90.4%	10	0	29
Vanderschueren	32	84.38%	2	0	72

M = major; m = minor.

**Table 2:** MWA treatment.

Author	N. of Procedures	Success Rate	N. of Complications	Follow-Up (Months)
Basile	7	100%	0	5–13
Rinzler	24	100%	4 (minor)	1
Kostrzewa	10	100%	0	6
Prud’homme	13	92.3%	3 (minor)	1
Reis	15	92.5%	1 (Major), 2 (minor)	33.8

M = major; m = minor.

**Table 3:** Cryoablation (CA).

Author	N. of Patients	Success (%)	Follow-Up (Months)	Recurrences	N. of Complications
Whitmore	29	90.5	12	1	6 m
Coupal	10	100	24	0	0
Santiago	21	100	21	0	3 m
Wu	6	100	28.7	0	1 m

M = major; m = minor.

**Table 4:** Interstitial laser ablation treatment (ILA).

Author	N. of Patients	Success Rate	Follow-Up (Months)	Recurrences	Complications
Gangi	114	99.1	58.5	6	1 m
Roqueplan	100	94	24	3	1 M, 3 m
Etienne	35	94.3	40	2	4 m
Witt	23	100	15	5	3 m
Tsoumakidou	57	100	12	1	0

M = major; m = minor.

## Data Availability

Data available in a publicly accessible repository.
